# The effect of social balance on social fragmentation

**DOI:** 10.1098/rsif.2020.0752

**Published:** 2020-11-18

**Authors:** Tuan Minh Pham, Imre Kondor, Rudolf Hanel, Stefan Thurner

**Affiliations:** 1 Section for Science of Complex Systems, Medical University of Vienna, Spitalgasse 23, 1090 Wien, Austria; 2 Complexity Science Hub Vienna, Josefstädter Straße 39, 1080 Wien, Austria; 3 London Mathematical Laboratory, 8 Margravine Gardens, Hammersmith, London W6 8RH, UK; 4 Santa Fe Institute, 399 Hyde Park Road, Santa Fe, NM 87501, USA; 5 IIASA, Schlossplatz 1, 2361 Laxenburg, Austria

**Keywords:** opinion formation, coevolutionary dynamics, social balance, phase transitions, social fragmentation, social cohesion

## Abstract

With the availability of internet, social media, etc., the interconnectedness of people within most societies has increased tremendously over the past decades. Across the same timespan, an increasing level of fragmentation of society into small isolated groups has been observed. With a simple model of a society, in which the dynamics of individual opinion formation is integrated with social balance, we show that these two phenomena might be tightly related. We identify a critical level of interconnectedness, above which society fragments into sub-communities that are internally cohesive and hostile towards other groups. This critical communication density necessarily exists in the presence of social balance, and arises from the underlying mathematical structure of a phase transition known from the theory of disordered magnets called spin glasses. We discuss the consequences of this phase transition for social fragmentation in society.

## 1. Introduction

Social cohesion and social fragmentation are central topics in the organization and functioning of large-scale societies. As such, these concepts have a long history of study [1–3]. Concerns have been raised that societies might be gradually losing their cohesion and becoming more fragmented due to ongoing changes in recent decades [4]. This might come with numerous catastrophic consequences such as riots, civil wars, governmental shutdowns, or even the decline of democracy [5]. In order to avoid these undesired outcomes, it is, therefore, necessary to understand the mechanisms leading to social fragmentation [6]. There exist two main approaches towards this problem: one regards fragmentation as an overall organization of a social network emerging through the evolution of its social ties, and the other considers it as the formation of clusters of similar social actors based on their preferences.

**Figure 1.**
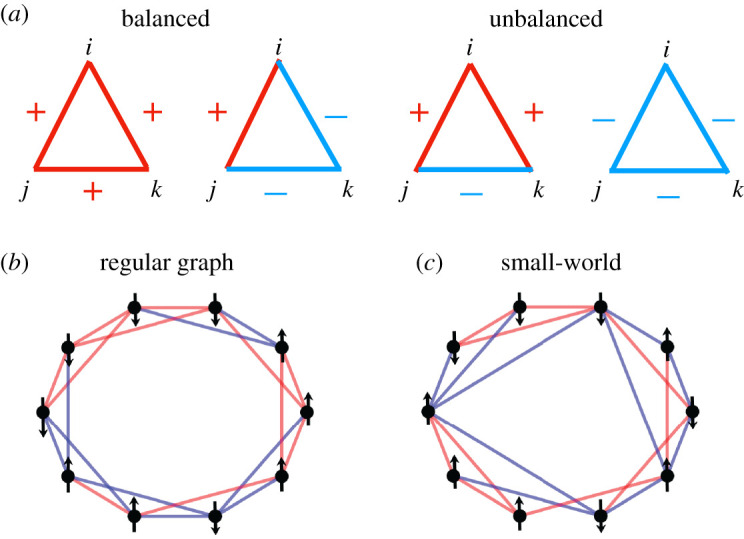
Balanced and unbalanced triangles (*a*). Red lines denoted with a plus sign represent friendly and cooperative relations between individuals *i*, *j* and *k*. Blue lines (minus) are negative or hostile links. A link between node *i* and *j* is denoted by *J*_*ij*_. It can be *J*_*ij*_ = +1 or *J*_*ij*_ = −1. A triangle is called balanced, if the product of its three-link states is *J*_*ij*_ *J*_*jk*_ *J*_*ki*_ = 1, and unbalanced, if the product is *J*_*ij*_ *J*_*jk*_ *J*_*ki*_ = −1. The first two triangles are balanced, while the second two are not. Network structure of our model society (*b*) and (*c*). Nodes represent individuals who have binary opinions that are displayed as either ↑ or ↓. Individuals are either linked by positive (red) or negative (blue) social ties. (*b*) A regular network topology, i.e. every node has the same number of neighbours; here *k* = 4. In (*c*), nodes are linked to others in a small-world network that can be obtained from (*b*) by randomly rewiring one side of any link with probability of *ε* = 0.2. Not everyone has the same number of neighbours anymore.

The first approach is based on the so-called structural balance theory. This theory is rooted in Heider’s concept of *balanced* and *unbalanced* triadic groups of individuals (triangles) [7].^1^ The former is created among either three mutual friends or two friends who have the same enemy. The latter emerges if, the three are mutual enemies or if two of them are enemies but the third is their mutual friend, see figure 1*a*. If an unbalanced situation occurs, individuals strive to release tension by flipping the sign of one of the three links, resulting in a balanced arrangement. Heider’s balance was later extended to a global balance, where a society is strongly (weakly) balanced^2^ if it can be partitioned into two (multiple) mutually antagonistic groups [8–10]. The evolution of social networks towards a balanced state has been shown in [11–22]. For an extensive review on the use of social balance theory in sociology, social psychology and anthropology, see [23], for a list of its empirical evidence, e.g. [24–29] and for evidence from neuroscience, see [30].

In the second approach, fragmentation emerges from agents' preferences [31]. Mostly, it is attributed to homophily, i.e. the tendency of people to form homogeneous groups sharing similar ethno-cultural, economical, or political values [32,33]. Other explanations were given by the ‘bounded confidence’ assumption [34,35], or by threshold opinion-adopting processes [36]. Finally, fragmentation may also arise if individuals coevolutionarily rearrange their social ties in response to changes in their ‘states’ [37,38]. Recent attempts have been made to impose social balance *a priori* as a constraint on the dynamics of agent states [39], or to drive the network towards balance by a change in the states, but not by the elimination of social tension in unbalanced triads [40–42].

Current approaches are limited by independently considering the effects of homophilous agent choices and that of social balance or by assuming that one implies the other. It remains unclear, however, what would happen to society if these two effects were to occur simultaneously and adaptively. While this question has been recently addressed in a number of works [43–48], a unified modelling framework able to quantify the *combined* effect of social balance and opinion dynamics has not yet been developed.

In this paper, inspired by a spin-glass approach towards coalition formation [49–52], we propose a stochastic, co-evolutionary model for social fragmentation where individuals’ opinions and the states of their social links co-evolve to minimize the system’s overall stress. In contrast to other models, social balance is explicitly taken into account via triadic interactions and is combined with agent choices by a mechanism of coevolution, rather than being imposed *a priori* or emerging from the dynamics of the social network alone. Further, while models based on social balance often consider *complete* networks, here we focus on more realistic *sparse* graphs where fragmentation develops as a function of the average connectivity in the society. We find a fundamental regime shift (phase transition) that happens at critical values of social connectivity. Below the critical density, we observe a largely cohesive society, where there is a sufficient density of positive links between groups. Above it, there exists an unavoidable phase that is dominated by the existence of many small internally cooperative communities, that are hostile and disagreeing towards other groups. Our main result supports the idea that the increasing level of fragmentation in recent years may be strongly related to the drastic rise of social connectedness due to technological advances, such as social media [53]. It should be noted, however, that there are claims in the empirical literature that provide evidence for both, the growth of polarization and its lack or even decrease, see [54–58].

## 2. The model

### 2.1. A coevolutionary model of opinion and social network formation

We assume that a society consists of *N* individuals that are labelled by latin indices, *i*. Each of these individuals is embedded in a social network and has social relations to *k*_*i*_ fellow individuals *j*. Since we are interested in the effect of the level of interconnectedness on social fragmentation, we keep the average number of links per person $k={\bar{k}}_{i}$ as a time-independent parameter of the model. Each relation between *i* and *j* can be either positive, *J*_*ij*_ = 1, if they are friends, or negative, *J*_*ij*_ = −1, if they are enemies. If two individuals are linked with a negative link this indicates a certain level of social stress. Each individual is endowed with an opinion, *s*_*i*_. For simplicity, we assume that there exists only one type of binary opinion, of the type: yes or no, Trump or Biden, etc. In figure 1, we show a schematic picture of our model society in the simplest case, where a total of *N* individuals with opinions (↑ and ↓) are linked to *k* neighbours each in a regular network (*b*) and in a so-called small world network [59] (*c*) with the same average connectivity, *k*.

Imagine that two individuals are linked through a positive link and they have opposite opinions on a given subject. We assume that this will cause a certain amount of social stress in the system. If, on the contrary, the two individuals do not like each other, *J*_*ij*_ = −1, and they have opposite opinions, this will not lead to stress. Both the opinions and the quality of the social links can be updated. Whenever *i* changes her opinion, we have *s*_*i*_ = 1 → *s*_*i*_ = −1, or *s*_*i*_ = −1 → *s*_*i*_ = 1. The same is true for social links, whenever we change friendship to enmity, *J*_*ij*_ = 1 → *J*_*ij*_ = −1, or vice versa. Assuming that on average, individuals tend to update their opinions and social links such that they reduce social stress, denoted by, *H*, we can formulate a simple stochastic coevolutionary model.

### 2.2. Minimizing social stress: a Hamiltonian approach

The system under study evolves to minimize overall social tension, which can be defined as

2.1 $$H=-\sum_{\left(i,j\right)}J_{ij}s_{i}s_{j}\;\;-g\;\sum_{\left(i,j,k\right)}J_{ij}J_{\;jk}J_{ki},$$

where *s*_*i*_ ∈ { − 1, 1} denotes the opinion of an individual *i* and *J*_*ij*_ ∈ {1, − 1} represents friendship and enmity between two connected agents *i* and *j*, respectively (*J*_*ij*_ = 0, if they are not linked). This type of cost function is called a *Hamiltonian* function in physics, where it captures the total energy in a system as a function of its configuration, *H* = *H*(*s*_*i*_, *J*_*ij*_). There it is used to implement the principle of minimization of energy.

**Figure 2.**
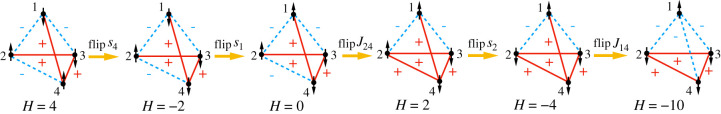
Example for how social stress, *H*, is gradually lowered in a sequence of changes of opinions and links between four individuals (assuming *g* = 1 for simplicity). Opinions of the nodes are given by ↑ and ↓, positive links are red, negative are blue. In the course of this sequence, the number of balanced triangles changes from two to four. Note that in the second step social stress is temporarily increased. This is a consequence of the stochastic nature of the model, where also unfavourable events happen from time to time.

In equation (2.1), the first sum describes the opinion adoption process between interacting agents (homophily term). It captures that individuals tend to act in a way as to avoid cognitive dissonance among them: if *i* and *j* are friends, they are more likely to share the same view, otherwise, they may hold opposing opinions. For any individual *i*, the simultaneous influences from all its neighbours *j* are represented by the sum over *j* of (*J*_*ij*_*s*_*j*_)*s*_*i*_ terms. The second term explicitly takes care of Heider’s social balance: it incorporates the tendency of suppressing unbalanced triangles between individuals. This effect is implemented by the sum over *all* possible triadic relations between any three individuals *i*, *j* and *k*. If *J*_*ij*_*J*_*jk*_*J*_*ki*_ = 1, they feel no social tension, otherwise social balance pushes them to change relations. Note that a link between *i* and *j*, *J*_*ij*_, in general will belong to several triangles. A flip of *J*_*ij*_ that lowers the *total* number of unbalanced triads should happen with higher probability than a flip that leads to an increase of unbalanced triangles, i.e. increases overall social stress. See the next subsection for how this is implemented. The parameter *g* in equation (2.1) controls the relative strength of the social balance term with respect to the opinion formation contribution (homophily term). In accordance with Heider’s theory, *g* must be positive so that balanced triangles do indeed dominate the unbalanced ones.^3^ Figure 2 shows an example for how four individuals with given initial opinions and links can change social stress, *H*, by flipping either opinions or links in order to reach a relatively stress-free situation, *H* = −10, that is socially balanced.

### 2.3. A stochastic coevolutionary model: the Metropolis algorithm

The social stress function, *H*, specifies the way in which the dynamical variables, *s*_*i*_ and *J*_*ij*_, tend to change over time. Assuming that humans generally tend to reduce social stress, changes that decrease *H* are favoured over those increasing it. This assumption implies that agents act as if they knew the system’s total stress–which, of course, may not hold in general. On the other hand, it leads to the prediction that the overall pattern of social relations must emerge as a minimum of the social stress. The minima, however, may not be reached if individuals make their choice locally without the knowledge of the whole system. In this sense, the possible outcome of a local dynamics would be less predictable compared to that obtained with our model.

We implement the simultaneous evolution of opinions and links by the Metropolis algorithm, a standard procedure in physics to minimize the system Hamiltonian [63]. In social network analysis, this optimization scheme is used to generate a time series of exponential random graphs *G*, whose stationary distribution is given by e^*H*(*G*)^, with *H*(*G*) the graph Hamiltonian. At this point, we distinguish two cases: opinions can change faster or slower than social links. We introduce a parameter *n* to control the *relative update rate* of opinions and links. For a society in which opinions update faster than links, the relative frequency of opinion updates versus link updates is chosen to be *nN*, with *nN* ≫ 1. Starting from a random configuration of opinions and links, the society is updated from one time step *t* to the next as follows:

1. Compute *H* of the current state of the system, assume it has a value of *H*_0_.
2. Pick a node *i* at random and flip its opinion, *s*_*i*_. Compute *H* again, it is now *H*_1_. If the value of *H* has decreased in response to the flip, *H*_1_ ≤ *H*_0_, accept the flip. If the value increased, accept the flip with probability, *p* = e^−Δ*H*/*T*^, where Δ*H* = *H*_1_ − *H*_0_ is the difference of stress before and after the flip. *T* denotes the ‘social temperature’ and is a model parameter. Pick the next node randomly and continue until *N* × *n* opinion updates (*n* Monte Carlo iterations) have been performed.
3. Compute *H* of the system at this point, assume that it is now ${\tilde{H}}_{0}$. We now pick one link randomly, *J*_*ij*_, and flip it. Compute *H* again, and assume it is ${\tilde{H}}_{1}$, we accept the flip if ${\tilde{H}}_{1}\leq {\tilde{H}}_{0}$, and accept it with probability $p^{\prime }={\text{e}}^{-\Delta \tilde{H}/T}$, where $\Delta \tilde{H}={\tilde{H}}_{1}-{\tilde{H}}_{0}$, if ${\tilde{H}}_{1}>{\tilde{H}}_{0}$. For simplicity, we assume that *T* is the same as in step 2.
4. Continue with the next time step.

Depending on the choice of *n* (and depending on the initial conditions), the opinions may or may not be given enough time to converge towards a steady state between link updates; in other words, they may or may not have enough time to ‘equilibrate’. In electronic supplementary material, figure S3, we show the consequences of different choices of *n*, keeping *n N* ≫ 1. We set *n* = 1 in the main body of the paper, in order to guarantee a true coevolutionary dynamics for the range of *N* considered. Appropriate care needs to be taken to ensure that the coevolution is correctly implemented when for larger systems deep valleys may develop in the ‘stress-landscape’ that can trap the system for a long time. In the electronic supplementary material, we consider the case *nN* ≤ 1, where link updates happen faster than the opinion updates, and occur before them.

The parameter *T* is a so-called *social temperature* that characterizes the average volatility of individuals in a society [64]. The higher *T*, the more ‘erratic’ or ‘irrational’ is an individual on average. This means that he or she is more likely to update his/her opinion and social ties, regardless if they reduce social stress. The update rules specified by *p* and *p*′ are based on the intuition that a change that reduces social stress (lower *H*) is more favourable than one that increases it. The choice of an exponential function is for convenience only and has no particular meaning (as it has in physics).

### 2.4. Social coherence through external influences

Opinion formation is not a purely endogenous process. It can be influenced strongly by external influences, such as religion, nationalism, and so on. Within the proposed framework, such influences can be included with additional terms in the *H* function. As a means to recover social cohesion, we propose to study a term that discourages people from maintaining hostile links. This could be the message of an exogenous religious or moral norm (love thy brother), or some nationalist propaganda that suggests that people of the same nation should be unconditionally friendly to one another. To this end, whenever we want to model an exogenous pro-social bias, we add a third term, $\left(h/2)\sum_{\left(i,j\right)}(1-J_{ij}\right)$, to equation (2.1). We consider *h* > 0, for which negative links are suppressed in the society.

### 2.5. Characterizing modes of collective behaviour: order parameters for social fragmentation

In the following, we are interested in a broad and generic definition of social fragmentation, closely following social balance theory: we call a society *fragmented* if there are many groups that are locally collaborative with a high density of ‘positive links’ within the group, but are often hostile to other groups. We call such groups ‘positive clusters’.^4^ On the other hand, a society is *cohesive* if one finds a sufficient density of positive links also between groups, such that one can ‘travel’ from group to group, without ever having to use negative links. In other words, in a cohesive society, groups are not necessarily hostile to each other if the positive links percolate. To characterize the degree of social fragmentation, we have to define appropriate quantities that we call *order parameters*. In the theory of phase transitions, order parameters signal regime shifts from one phase to another. To quantify the degree of social fragmentation, we use the following measures:

#### 2.5.1. Size distribution of echo chambers

A signal for social fragmentation is the distribution of positive cluster sizes. We detect positive clusters, following a standard network partitioning procedure, where an objective function equal to the total number of positive relations between clusters and negative links within them is minimized. This approach makes use of the notion of the line index of balance that was first proposed in [66] and subsequently developed in the framework of the signed blockmodel [67]. The line (deletion) index of balance measures the minimum number of links whose removal results in balance.

In the optimal partition, most of the negative links will be found between the positive clusters, and only a small fraction of negative links remains within them [68]. A better partition for real social networks can be obtained if one allows for clusters of only negatively linked agents [68]. As such ‘negative clusters’ are not used for our definition of fragmentation, we do not use this later approach here. Finally, among the detected ‘positive’ clusters, we select those that consist of only like-minded agents and define them as ‘echo chambers’. The size of an echo chamber is thus given by the number of such nodes, and is denoted by S(E), where E denotes the chamber.

#### 2.5.2. A measure for polarization, *f*

We introduce a simple network variable, *f*, to measure the level of social balance in the society. It is defined as the difference of the fractions of balanced and unbalanced triangles in the network

2.2 $$f=\frac{n_{+}-n_{-}}{n_{+}+n_{-}},$$

where *n*_+_ and *n*_−_ are the number of balanced and unbalanced triangles, respectively.^5^ *f* = 1 means that all triangles are balanced, *f* < 1 signals that unbalanced triangles are present. Even though *f* could be negative, this situation is never observed in simulations. This is in agreement with both Heider’s intuition and the empirical evidence obtained in real social networks, where the value of *f* is typically above 0.7 [25]. The case *f* → 0 corresponds to an equal number of balanced and unbalanced triangles. One can show that if the network is balanced (clusterable), i.e. it can be partitioned into two or multiple clusters, within which *all* links are positive and between which links are *exclusively* negative, then all triangles are balanced, i.e, *f* = 1 [65]. Conversely, while the case, *f* = 1, is sufficient to imply such a partition for complete networks; it is just a necessary, but not a sufficient condition for a *sparse* network to be clusterable. In the literature, a graph in which all triangles are balanced (*f* = 1) but cycles of longer length are not, is called *limited clusterable* [69]. For this kind of graph as well as for those having most of triangles balanced (*f* → 1), a partition which is close to that of clusterable networks, generally exists.

#### 2.5.3. A measure for social tension, *e*_*f*_

As society tends to reduce social tension over time, the lowest value of stress can be achieved at low ‘social temperatures’. We can use the ratio between the system stress of the current state, *H*, and the absolute value of the ground state |*H*_ground state_|, to indicate the relative level of social stress. Let *e*_*f*_ denote this ratio. By definition, *e*_*f*_ ≥ −1. It is equal to −1 if and only if no tension remains in the society, while higher values of *e*_*f*_ are observed at higher temperature, where unbalanced triangles necessarily exist. A similar stress-based measure has been used previously to quantify the level of social balance in large-scale social networks [26]. Note that the ground state of the system in equation (2.1) is highly degenerate, i.e., there exist many states with the lowest possible stress level. The presence of an external influence *h*, can modify the ‘stress-landscape’, resulting in the emergence of global cooperation or consensus among individuals.

#### 2.5.4. A measure for opinion diversity, *m*

As a simple measure for the opinion diversity across groups, we compute the overall opinion of the society,

2.3 $$m=\frac{1}{N}\left|\sum_{i}^{N}s_{i}\right|.$$

By definition, *m* ∈ [0, 1]. The lower *m* is, the more diverse opinions are. Opinions are aligned across society if *m* → 1. This measure can also serve as a probe of how fast opinions converge to a consensus. This is important because in real social contexts one can change opinions and friends (or enemies) within a limited lifetime. Therefore, convergence times do matter and must be studied in detail. The time required for the system to equilibrate from different initial conditions may vary strongly.

## 3. Results

We simulate the model given in equation (2.1) for the parameter choices of *N* = 400, and *g* = 1. We first discuss the phase diagram of the model and its consequences.

### 3.1. Phase diagrams

**Figure 3.**
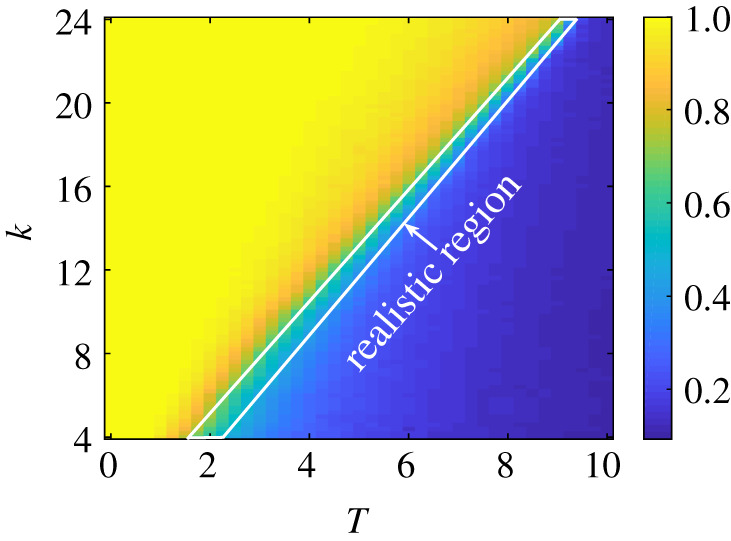
Phase diagram of the stochastic coevolutionary model with social balance. The balance level, *f*, is shown as a function of the average network degree, *k*, and social temperature, *T*. A phase separation line is visible. Below it, for low values of connectivity and high *T*, there exits a socially coherent phase (blue), above the line there is a phase of social fragmentation (yellow). Empirically reasonable values of *f* around 0.7 are indicated with a white box. Results were obtained for regular graphs (*ε* = 0), *g* = 1, *N* = 400 and are averaged over 500 realizations. Random initial conditions in links and opinions.

The central result of this paper is shown in figure 3 that shows *f* (in colour code) as a function of the average connectivity, *k*, and social temperature, *T*. There is a clear separation line, $k_{c}(T)∼\alpha T$, at which the society transitions from a coherent situation with *f* ∼ 0.1 (blue) to a fragmented one, characterized by *f* ∼ 1 (yellow). In the yellow region, the emergent networks are strongly balanced and opinion clusters exist. These polarized clusters disappear and opinions become randomly distributed among agents in the dark blue region, where there are as many balanced as unbalanced triangles. Note, that values of *f* ∼ 0 are unrealistic. Real societies are balanced and show empirical values in a range around *f* ∼ 0.7 [24,25]. We indicate the realistic region with a white box. Assuming that a given society is found somewhere in the realistic region, say at a fixed *T*, it only takes a small increase of social connectivity, *k*, for the society to be pushed into the fragmented phase. In recent years, the average connectivity has certainly increased in societies, making it easier for them to transition into the fragmented regime. The result in figure 3 is obtained for a regular network (*ε* = 0). We confirmed that the existence of the separation line also holds for small-world network topologies and for adaptive networks with evolving topologies; see also below. This line does also exist in the case, where link updates happen much faster than that of opinions, see electronic supplementary material, figure S4.

### 3.2. Size distribution of echo chambers

**Figure 4.**
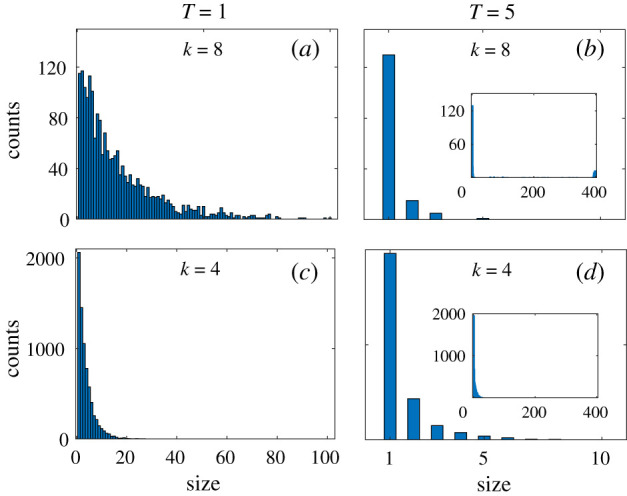
Distribution of echo chamber sizes, S(E), as a function of average connectivity, *k*, and social temperature, *T*. Echo chambers are defined as groups of friendly agents who hold the same opinion. The left column represents the situation in the fragmented phase (low temperature *T* = 1). The right column is in the cohesive phase. The upper panels show an average connectivity of *k* = 8, the lower ones *k* = 4. In the fragmented phase, there appear significant groups of all sizes that are characterized by uniform opinions and positive relations within, and hostile relations towards others. The insets show the size distribution of the detected ‘positive’ clusters, $C_{k}$. These are groups of cooperating individuals, where any two members of the same group can be connected by a path consisting of positive links only. In the cohesive phase, there may exist positive clusters of maximal sizes ($S(C_{k})=400$), meaning that the whole society can cooperate despite a diversity in opinions. Same model parameters as in previous figure. Results were obtained from 500 realizations.

We show the echo chamber size distribution for various values of *T* and *k* in figure 4. The left column shows the situation for low social temperature, *T* = 1, the right column shows a high temperature *T* = 5. The upper panels show a high average connectivity, *k* = 8, the lower ones correspond to *k* = 4 neighbours. The left column corresponds to the fragmented phase, the right to the cohesive phase. Deep in the fragmented phase, we observe a broad distribution of echo chamber sizes, spreading to sizes of about 100 for *k* = 8 and to sizes of about 20 for *k* = 4. In the right column, there are sharply peaked distributions with maximum cluster sizes of about 2–3, meaning that there are no large clusters of unique opinion forming. This corresponds to a society where different opinions coexist. The insets show the size distribution of the ‘positive’ clusters, $C_{k}$, found by the mentioned partitioning method. Note that in (*b*), there is a small peak at 400, which is the maximal size of a cluster. This indicates the possibility of global cooperation of the whole society in the cohesive phase even if opinions are diverse.

### 3.3. Robustness

To confirm that results are robust with respect to changes of parameters, we perform a series of robustness checks.

**Figure 5.**
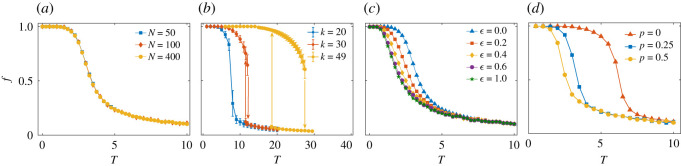
Robustness of results. (*a*) Size dependence. Section of the phase diagram for regular networks with *k* = 8 for various sizes of society, *N* = 50, 100, 400. No size dependence in the phase diagram is visible. (*b*) Higher average connectivity pushes the phase transition towards higher critical temperatures. A discontinuous transition of *f* is observable that shows a hysteresis effect. Note that it is especially pronounced for large connectivities. The existence of this hysteresis could indicate a potential handle to avoid fragmentation, see discussion. *N* = 50, results are averaged over 5000 independent realizations of the model. (*c*) Change of the phase transition for a small-world network structure with *ε* = 0, 0.2, 0.4, 0.6, 1. *k* = 8, *N* = 400, *g* = 1. Results averaged over 200 realizations for every *ε*. (*d*) Dependence of the phase transition on a random rewiring process, where in addition to the flip of the links’ sign according to social balance, one also considers the possibility of breaking a link with probability *p*, and then creating another one between two randomly chosen individuals. These new links can be either positive or negative, with the same probability 1/2. Here, *p* = 0, 0.25, 0.5, *k* = 16, *N* = 400, *g* = 1, and results are averaged over 200 realizations for every *p*.

We first test the dependence on the size *N* of the society. For a fixed value of *k* = 8, we show a section of the phase diagram in figure 5*a* for various system sizes, *N* = 50, 100, 400 on regular networks. Within the range of *N*s considered, no size dependence is observed. The lack of size dependence could suggest that the result may still hold in the limit *N* → ∞ for this type of network topology.^6^

In figure 5*b*, we show the effect of the average connectivity on the results, where we fix *N* = 400 and compute *f* for various values of *k*. As already noted in figure 3, with increasing *k*, the phase diagram is shifted towards the fragmented phase (yellow region in the phase diagram). The transition appears to be discontinuous (first-order), meaning that *f* jumps as a function of the social temperature. Figure 5*b* also demonstrates a hysteresis effect (visible for *k* = 30), which often accompanies first-order transitions. This can be understood in the following way: if in figure 5*b*, we increase *T*, *f* starts to gradually decrease, and then drops rapidly to much lower values. If at that point one would start decreasing *T*, *f* would not immediately jump up to previous levels, but remain low until at a lower *T* it would finally jump upward again. See arrows in the figure.

To test if the particular network structure has an influence on the results, we computed the phase diagrams with small-world networks [59]. The small-world parameter, *ε*, controls the probability to re-connect a link from any node to any other node. Here, we rewire the connections in such a way that the network remains connected and does not dissociate into different components. Note that *ε* = 0 means a regular network, *ε* = 1 corresponds to a random graph. Figure 5*c* shows the result. The transition is shifted towards lower temperatures, i.e. the critical temperature decreases with increasing *ε*. This fact can be understood as a consequence of having less triangles in the networks that are obtained with a larger value of *ε*, making social balance effectively weaker; see electronic supplementary material, figure S5.

Next, we check the effect of a time-evolving topology on the phase transition. Figure 5*d* shows a significant reduction in the critical temperature if random rewiring takes place. In this process, agents can either change the sign of a link with probability 1 − *p* or rewire with a new link with probability *p*. The new link, if created, can be either positive or negative with the same probability 1/2, regardless of agent opinions. For any fixed value of *k*, this process results in a lower critical point, *T*_*c*_. The higher the rewiring rate *p*, the lower is *T*_*c*_. However, for a given value of *p*, we observe that social fragmentation extends with growing connectivity, *k*, see the electronic supplementary material for details. The main result of figure 3 is also confirmed in this case. In the electronic supplementary material, we also consider another rewiring scheme that can establish a new link whose sign is not chosen randomly, but is determined by the agents' opinions: it is positive if two agents are similar or negative otherwise. We call this mechanism homophilious rewiring. The result remains qualitatively the same as in the random rewiring case, namely *T*_*c*_ increases with *k*, for fixed *p*. However, while large random rewiring (*p* → 1) significantly lowers *T*_*c*_, homophilious rewiring results in a higher *T*_*c*_ even in the absence of social balance (*p* = 1).

Finally, we check what happens if we *explicitly* lower the coupling strength of the Heider term in equation (2.1). When we take *g* = 0.01, we observe a shift in the phase transition line to the left and the dependence of the transition on the connectivity *k* becomes negligible; see electronic supplementary material, figure S1. Obviously, in the limit *g* → 0 where the Heider term vanishes, there will be no more dependence on *k*. The pronounced fragmentation transition at high interconnectedness is hence a direct consequence of social balance, in the absence of which society remains cohesive unless the social temperature becomes very low.

### 3.4. The role of external influences

**Figure 6.**
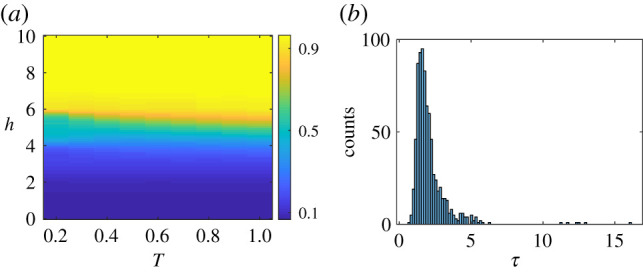
(*a*) Opinion diversity (colour), *m*, as a function of social temperature, *T*, and the external influence parameter, *h*. The blue region indicates the case where opinion clusters exist. In the yellow region, global consensus is the unique attractor of the dynamics. Note the change of meaning of yellow and blue with respect to figure 3. Note that the formation of global alignment of opinions takes very long (*O*(10^4^) Monte Carlo iterations). *N* = 200, *k* = 10, *g* = 1, *ε* = 0, results averaged over 3200 realizations. (*b*) Distribution of convergence times, *τ*, at *T* = 1. Results were obtained for regular networks *ε* = 0, *g* = 1, *N* = 200, *k* = 10 and are averaged over 800 realizations. *τ* is measured in the unit of *kN* time steps, each consists of *n N* attempts to flip opinion. Its average is $\bar{\tau }$ ≃ 2.11 × *kN*, its variance is $\sigma_{\tau }^{2}≃1.3\times kN$.

In figure 6*a*, we show the effect of the external influence, *h*, designed to suppress negative links in the society, on the fragmented phase. It does what it is expected to do. Note that the terms *h* and *g* may compete with each other: if *h* promotes the flip of a negative link this could result in more unbalanced triangles, meaning that it works against the effect of *g*. As a consequence of this competition, a low value of *h* can only remove a small fraction of negative links and a fragmented society emerges, similar to the case without the external influence. Only beyond a critical threshold, *h*_*c*_, can most of the negative links be eliminated and global consensus be reached. See electronic supplementary material, figure S8 for an illustration of this phenomenon for a simple network of *N* = 3 nodes. Since there is no transition in *f* (it remains close to 1), we use *m* to characterize the change in the final state of the society under the effect of the external influence.

### 3.5. A note on timescales

In figure 6*b*, we analyse the times, *τ*, that are necessary for the order parameters to converge to their stationary values. This is essential to check since convergence times for this type of system can be exceedingly (and thus unrealistically) long. *τ* is the time required for the system to equilibrate at low social temperature. We observe in figure 6*b* that on average *τ* is of the order of *kN* timesteps. Given that the number of links is *kN*/2, the distribution of *τ* with a mean of 2.11 × *kN* means that the network updates about four times on average before reaching equilibrium. However, for a particular run, *τ* may vary substantially, depending also on the initial conditions. Typically, the steady state can be reached faster if the initial fraction of positive links is above 1/2. The variability becomes more pronounced in the presence of an external influence, *h*. At sufficiently low temperature, the convergence times can become very long due to the existence of many local minima, so-called jammed states, in the stress landscape [13,17]. The social stress in a jammed state is not larger than that in any of its neighbouring states, which can be reached from this state by a single opinion or link flip. Evolving on such a ‘rugged landscape’, the system is very likely to get trapped in local minima. The global minimum of the social tension, *H*, hence may even become unobservable during simulation time.

## 4. Summary and discussion

We proposed a model that captures five key elements of human societies: (i) *Agency.* Humans make their decisions individually. (ii) *Social context—social networks.* Individuals are constantly influenced by opinions and actions of others in their social neighbourhood, or by other external influences. Humans tend to show homophily. (iii) *Stochasticity.* Individuals are not fully rational and take random decisions from time to time, that do not maximize utility functions. (iv) *Coevolution.* Individuals update their opinions as well as their social links. Most of these updates tend to avoid social tension. (v) *Social balance.* Social networks show robust structures in positive and negative social links. They exhibit patterns of social balance.

We implemented the model in a stochastic manner in the framework of a Hamiltonian approach. The focus of the model rests on the notion that humans tend to update opinions and social links, so as to reduce social tension. The model has a non-trivial phase diagram, i.e. it shows at which parameter values tipping points occur where society drastically changes its microscopic composition and structure.

The results deliver a clear and robust message: a society with the ability of a coevolutionary dynamics of opinion- and link formation must be expected to have a phase diagram as the one presented in figure 3. This is a direct consequence of the social balance term in the model, which incorporates the empirical fact that societies are socially balanced to a high degree. The phase diagram shows the existence of a critical connectivity, *k*_*c*_, between individuals of a society at a fixed social temperature, *T*, that controls the update frequencies of opinions and links. Below the critical connectivity, *k*_*c*_, society is in the cohesive phase, where opinions coexist. Above the critical connectivity, society fragments into clusters of individuals who share positive links within the clusters and have negative links between groups. Within the clusters, large patches of uniform opinions form, and a strong reinforcement of homophily is observed. The existence of a critical connectivity is an extremely robust phenomenon; if the connectivity increases above the critical value, society *must* fragment.

The model also gives answers to how the fragmented phase can be avoided. There are only two ways out: either to lower the connectivity below the critical density, *k*_*c*_, by reducing the number of interaction partners (social distancing) or, alternatively, to increase the social temperature, *T*, meaning that people would update their opinions (and links) randomly more often. There are no other alternatives within the framework of this model. For the case of increasing update rates, however, the existence of the above-mentioned hysteresis phenomenon must be taken into account. This means that if at a fixed interaction density, *k*, update rates, *T*, are increased, the fragmentation might transition rapidly to the mixed opinion phase, at, say *T*′. If then the update rates are again reduced, fragmentation does not immediately return, but might reappear at lower update rates, *T*″ < *T*′. The observed hysteresis seems to be a universal feature shared by dynamical models of social networks with Heider’s balance [61,62].

The position of the critical lines can be shifted if the strength of the Heider term *g* changes or if the network topology evolves in a rewiring process with rate *p*, or if the (fixed) underlying network becomes irregular, for *ε* > 0. However, the essence of the model, namely, the necessary existence of social fragmentation at high connectivity remains unaffected. The phase diagram remains robust with respect to changes in the overall size of the society. We have seen that under strong exogenous influences, *h*, such as religion or nationalism, there is a possibility of transitioning from a fragmented society to a ‘utopian’ or fascist one; such interventions will externaly force the society towards a global consensus.

The presented model has a number of shortcomings. Several essential features of real societies have not been included. We strongly simplified the structure of social connectivity. Whereas social systems are multi-layer networks, here we have focussed only on a single layer. It remains to be seen how the phase diagrams change under the integration of more than one layer of (positive and negative) social interactions—an approach recently proposed in [70].

The use of one single binary opinion is minimalistic and unrealistic. It would be more realistic to use multiple opinions such as cultural features in the Axelrod model [32]. The dynamics would have to be modified to account for negative links. For example, two agents connected by a positive link can become more similar after interaction, while those who are hostile to each other should drift further apart in the space of opinions. It would be interesting to compare the effect of social balance on the fragmentation in this case with the one that occurs in the presented model. The key message of our model should remain valid as the social balance ensures the existence of clusters of positive links, within each of which opinions are driven toward uniformity by the reinforcement effect of homophily, regardless of the opinion multiplicity. This picture, however, needs to be compared with a recent work [47] that demonstrates homophily may prevent society from reaching a social balance state.

The use of the same social temperature for both, the opinion and the link update, is not justified a priori and has been applied for the sake of simplicity. To describe situations, in which, either agents’ opinions are more frequent to change than their relations, or vice versa, we introduced the parameter, *n*. As shown in the electronic supplementary material, within a range of *n* that ensures a true coevolutionary dynamics, the results do practically not depend on *n*. Further, the structure of the phase diagram remains similar when links are updated faster than opinions, which implies that our main conclusion does not depend on details of the simulation scheme. Alternatively, a stochastic dynamics with two temperatures, one for opinions and one for links is certainly reasonable. However, in such a generalization, a more complicated non-equilibrium approach is required. The structure of the phase diagram may become richer with long-lived metastable phases. Such a non-equilibrium approach has been considered recently in [42], where network evolution is not driven by Heider’s balance, but by another aspect of cognitive dissonance. There, fragmentation emerges either as an absorbing steady state of the dynamics, or from an active phase due to fluctuations in systems of finite size.

Finally, from a technical side, the model presented here is a variation of a spin glass model used in physics. With the present choice of model parameters (low connectivity, networks of finite size, *n* = 1), we can not expect to find the complicated phase space structure of a mean-field spin glass [71]. However, the essence of frustration imposed by the Heider term is the same as in spin glasses. A more detailed technical study of the model is going to be published elsewhere.

## 5. Final conclusion

The main conclusion of this paper is that in the presence of social balance, fragmentation must occur in a coevolutionary society of homophilous individuals if the average interaction density exceeds a critical threshold.

## Endnotes

1 He also considered the P-O-X triple between two people *p*, *o* and an impersonal entity *x*, whose balanced and unbalanced states are defined in a similar way.

2 Weakly balanced networks are also called clusterable, following the clusterability theorem of Davis [8].

3 At this point, it is not clear how to empirically infer the value of *g* for a given society. We chose *g* ∈ [0, 1] in the model implementation. In fact, any non-negligible value, *g* ≠ 1, can be shown to yield similar results as *g* = 1 (see electronic supplementary material). This choice corresponds to the assumption that the effect of social balance is comparable in importance to the opinion terms (social influence) in equation (2.1). As long as the number of links and that of triangles are of the same order of magnitude (as is the case in sparse networks), it seems reasonable to keep the contribution of the Heider term comparable to that of opinions. In physics, the case *g* = 0 corresponds to the classical Edwards–Anderson spin glass model [60], while the other extreme, *g* → ∞, it corresponds to the model studied in [13,17,61,62].

4 These groups are closely related to the so-called cohesive subgroups in the social network literature [65].

5 Note that following Heider’s original notion of balance, we consider a triangle with three negative links as unbalanced, in contrast to Davis’ definition of weakly balanced triads, where this type of triangle is considered as balanced [8].

6 In this case, the relative update ratio of opinions to links must be modified appropriately.

## Data accessibility

This article has no additional data.
